# Percutaneous treatment of pulmonary vein stenosis: a transoesophageal echocardiography step-by-step guide in diagnosis and treatment

**DOI:** 10.1093/ehjimp/qyag083

**Published:** 2026-05-04

**Authors:** Gabriella Locorotondo, Annalisa Pasquini, Andrea Pica, Monica Filice, Piero Farina, Antonella Lombardo, Francesca Graziani, Cristina Aurigemma, Enrico Romagnoli, Carlo Trani, Massimo Massetti, Francesco Burzotta

**Affiliations:** Department of Cardiovascular Medicine, Fondazione Policlinico Universitario A. Gemelli, IRCCS, Largo Agostino Gemelli 8, Rome 00168, Italy; Department of Cardiovascular Medicine, Fondazione Policlinico Universitario A. Gemelli, IRCCS, Largo Agostino Gemelli 8, Rome 00168, Italy; Department of Cardiovascular Medicine, Fondazione Policlinico Universitario A. Gemelli, IRCCS, Largo Agostino Gemelli 8, Rome 00168, Italy; Department of Cardiovascular Medicine, Fondazione Policlinico Universitario A. Gemelli, IRCCS, Largo Agostino Gemelli 8, Rome 00168, Italy; Department of Cardiac Surgery, Casa di Cura ‘Villa Verde’, Taranto, Italy; Department of Cardiovascular Medicine, Fondazione Policlinico Universitario A. Gemelli, IRCCS, Largo Agostino Gemelli 8, Rome 00168, Italy; Department of Cardiovascular Medicine, Fondazione Policlinico Universitario A. Gemelli, IRCCS, Largo Agostino Gemelli 8, Rome 00168, Italy; Department of Cardiovascular Medicine, Fondazione Policlinico Universitario A. Gemelli, IRCCS, Largo Agostino Gemelli 8, Rome 00168, Italy; Department of Cardiovascular Medicine, Fondazione Policlinico Universitario A. Gemelli, IRCCS, Largo Agostino Gemelli 8, Rome 00168, Italy; Department of Cardiovascular Medicine, Fondazione Policlinico Universitario A. Gemelli, IRCCS, Largo Agostino Gemelli 8, Rome 00168, Italy; Department of Cardiovascular Medicine, Fondazione Policlinico Universitario A. Gemelli, IRCCS, Largo Agostino Gemelli 8, Rome 00168, Italy; Department of Cardiovascular Medicine, Fondazione Policlinico Universitario A. Gemelli, IRCCS, Largo Agostino Gemelli 8, Rome 00168, Italy

**Keywords:** pulmonary vein stent implantation, intraprocedural transoesophageal echocardiography, step-by-step guidance

## Abstract

**Aims:**

Acquired severe pulmonary vein stenosis (PVS) is a rare but clinically relevant complication of radiofrequency catheter ablation for atrial fibrillation. Diagnosis is often delayed or missed without a multimodality imaging approach. Once established, percutaneous intervention with balloon angioplasty or pulmonary vein stent implantation represents the preferred therapeutic strategy. Imaging plays a central role in determining the indication for intervention, assessing the severity and extent of disease, and accurately localizing the stenosis, thereby enabling appropriate procedural planning.

**Methods and results:**

This practical review provides a comprehensive overview of the role of multimodality imaging in patients with suspected PVS and proposes an integrated workflow for diagnosis, treatment, and follow-up. In addition, it offers a step-by-step illustrated guide to the use of transoesophageal echocardiography during percutaneous intervention, addressing the current lack of standardized protocols.

**Conclusion:**

The review is structured to cover diagnostic pathways, procedural guidance, and post-intervention surveillance.

## Graphical Abstract

**Graphical Abstract:**
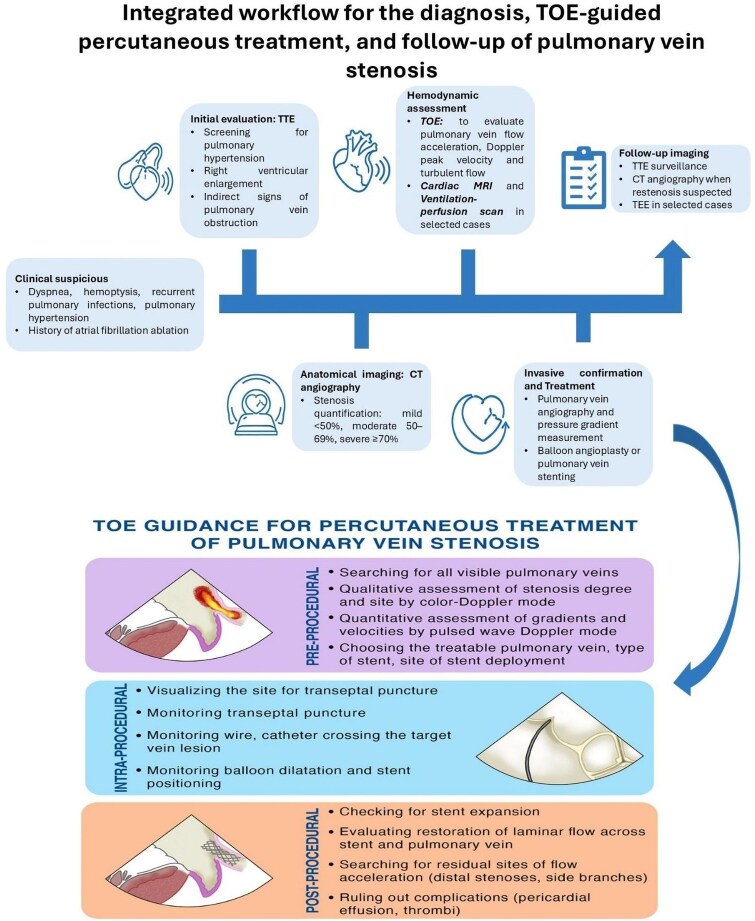
The diagram illustrates an integrated multimodality imaging and procedural workflow for the diagnosis and treatment of pulmonary vein stenosis. A step-by-step approach to the pre-, intra-, and postprocedural guidance of percutaneous stenting for pulmonary vein stenosis using transoesophageal echocardiography (TOE) is also proposed.

## Introduction

Acquired pulmonary vein stenosis (PVS) is a rare complication of radiofrequency (RF) catheter ablation of atrial fibrillation (AF). The incidence of PVS following AF ablation varies significantly according to the energy source and ablation technique.^1^ Early focal RF ablation approaches were associated with rates of PVS as high as 6.3%, whereas contemporary circumferential antral RF techniques have reduced this risk to approximately 1%.^2^ Cryoballoon ablation, particularly with second-generation devices, has been associated with even lower rates, generally below 1%.^3^ More recently, pulsed field ablation has demonstrated a highly favourable safety profile, with current evidence suggesting that severe PVS is virtually eliminated with this technology.^4^ The vascular damage from RF leads to a reduction of pulmonary vein (PV) diameter, which can also occur after Cryoballoon ablation.^5^ Mild pulmonary vein stenosis (PVS) is relatively common, whereas severe or symptomatic PVS—occurring in <1% of cases^6^ and is associated with significant morbidity.^7^ Without routine post-ablation screening, the diagnosis is often delayed or missed. The mean delay of PVS manifestation has been estimated to be 4 ± 3 months; symptoms include dyspnoea, cough, fatigue, exertional chest pain and haemoptysis.^8^ Failure to promptly diagnose and address this condition may result in the need for lung lobectomy with life-threatening risks.^9^

In this review, we provide an overview of the role of transoesophageal echocardiography (TOE) in the diagnosis and procedural guidance of balloon angioplasty (PBA) and pulmonary vein stent implantation (PSI) for PVS, the current treatment modalities.^10^ A recent meta-analysis suggests that PSI is associated with improved patency rates compared with PBA alone.^11^ PVS surgery is still an option for late presentations but requires cardiopulmonary bypass^12^; the percutaneous approach is the preferred one, provided that all efforts are made for no standardized procedures, and careful planning is required for reducing the risk of complications.^13^

Before catheterization and intervention, the contribution of imaging studies is essential for the indication to intervention, for assessing the severity and the number of the stenotic veins, for establishing the location of the stenosis, and for evaluating both the residual diameter of the most stenotic segment and the widest diameter in the upstream portion, considered as the reference diameter.^14^ All these evaluations allow a precise diagnosis of PVS and appropriate planning of the procedure.

## Diagnosis

### Multimodality imaging

Diagnosis is based on multimodality imaging studies, which include computed tomography (CT), cardiac magnetic resonance (CMR), echocardiography,^15^ and intracardiac echocardiography (ICE). Pulmonary vein stenosis is typically first suspected on transthoracic echocardiography (TTE), which serves as a screening tool by identifying indirect signs such as pulmonary hypertension or right ventricular dysfunction. TOE provides detailed visualization of the proximal pulmonary veins and haemodynamic information, and it plays a crucial role in procedural guidance. However, disability to assess distal segments is limited, and it remains an angle- and operator-dependent technique. For these reasons, CT angiography, including spectral-CT reconstructions that allow accurate visualization of the pulmonary vein lumen,^15^ represents the primary non-invasive modality for anatomical assessment, quantification of stenosis severity, and procedural planning, including stent sizing. Based on CT-derived luminal narrowing, PVS is classified as mild (<50%), moderate (50–69%), or severe (≥ 70%).^16^ CMR offers a radiation-free alternative and can provide additional perfusion information.^17^ Ventilation-perfusion scan can help in assessing PVS severity and its haemodynamic impact, especially in borderline cases.^8^ ICE with 2D and 3D reconstructions can provide detailed anatomical information of the proximal PVs, and RF-induced tissue changes in the PV wall^18^; it may represent a valuable alternative to TOE for procedural guidance in selected patients, particularly when general anaesthesia is to be avoided or TOE is contraindicated. It can be performed without oesophageal instrumentation; however, it has a smaller field of view, requires additional venous access, provides limited Doppler assessment, and remains operator dependent. Invasive cardiac catheterization is required for definitive haemodynamic confirmation of pulmonary vein stenosis: the classical finding is an elevated mean pulmonary vein pressure (PCW pressure) with a pulmonary vein (PV) to left atrium (LA) gradient > 3 mmHg. There is also loss of phasic flow, which corresponds to decreased pulsatility at Doppler examination.^19^ The confirmation of PVS requires venography, pulmonary artery wedge angiography, and measurement of the actual pressure gradient between the PV and the LA.^14^

*Tables 1* and *2* summarize each imaging modality and the key diagnostic findings of PVS.

**Table 1:** Multimodality imaging for pulmonary vein stenosis assessment

Imaging modality	Main role	Strengths	Limitations	Key diagnostic findings
**Transthoracic echocardiography (TTE)**	Initial screening	Widely available, non-invasive, repeatable	Limited direct visualization of pulmonary veins	Indirect signs: pulmonary hypertension, right ventricular dilation
**Transoesophageal echocardiography (TOE)**	Detailed assessment of proximal pulmonary veins and procedural guidance	High spatial and temporal resolution, Doppler haemodynamic assessment	Limited visualization of distal segments, angle dependency	Increased Doppler velocity (>1.1–1.5 m/s), turbulent flow
**CT angiography**	Reference technique for anatomical assessment	Excellent spatial resolution, accurate luminal measurement, and stent sizing	Radiation exposure, contrast use	Direct visualization of luminal narrowing and stenosis severity
**Cardiac magnetic resonance (CMR)**	Alternative anatomical and functional assessment	No radiation, perfusion evaluation possible	Lower spatial resolution vs. CT, longer acquisition	Reduced pulmonary vein diameter, perfusion asymmetry
**Ventilation/perfusion scintigraphy**	Functional evaluation	Quantifies regional pulmonary perfusion	Limited anatomical information	Reduced perfusion in affected lung segments
**Invasive venography with pressure gradient measurement**	Hemodynamic confirmation	Gold standard haemodynamic assessment	Invasive	Pressure gradient across stenosis

The table summarizes the main role, strengths, limitations, and key diagnostic findings of the several imaging techniques that are useful for pulmonary vein stenosis diagnosis.

**Table 2:** Key diagnostic findings suspicious for pulmonary vein stenosis

Parameter	Normal finding	Suspicious for PVS	Pathophysiological explanation	Limitations
**Peak pulmonary vein Doppler velocity (TOE)**	<0.8–1.0 m/s	>1.1–1.5 m/s	Flow acceleration across a narrowed lumen according to the continuity principle	Angle dependency, influenced by flow volume
**Flow turbulence at colour Doppler (TOE)**	Laminar flow	Colour Doppler turbulence at PV ostium	Flow disturbance due to stenosis	May occur with high flow states
**Spectral Doppler pattern (TOE)**	Biphasic systolic–diastolic pattern	Monophasic continuous high-velocity flow	Loss of normal atrial pressure modulation	May be altered by atrial pressure changes
**Pulmonary vein diameter (TOE/CT)**	Normal calibre	Reduced lumen diameter	Anatomical stenosis	Echocardiographic measurement limited
**Pressure gradient (invasive)**	Minimal	Elevated gradient across stenosis	Haemodynamic obstruction	Requires invasive assessment
**Associated findings**	Normal pulmonary pressures	Pulmonary hypertension, RV dilation	Chronic venous obstruction	Non-specific

CT: computed tomography; TOE: transoesophageal echocardiography.

### Transoesophageal echocardiography

TOE is useful in identifying and confirming PVS, in determining pressure gradients and for procedural guidance. PVs evaluation by TOE can be difficult due to their arise from the roof of the LA, and thus close to the transducer; they cannot be displayed all at once, not even by using three-dimensional (3D) reconstructions.^20^ Usually, four PVs enter LA: left PVs being located postero-laterally, and right PVs running behind the superior vena cava (SVC) and the right atrium (RA) and reaching the LA supero-medially, near the interatrial septum (IAS). However, PV anatomy may differ in up to 38% of the normal population.

The standard TOE approach for visualization of PVs requires scanning from the mid-oesophageal (ME) level. The standard views allow a better visualization of the upper (superior) PVs, while the lower (inferior) branches may not be adequately visualized.

**Figure 1:**
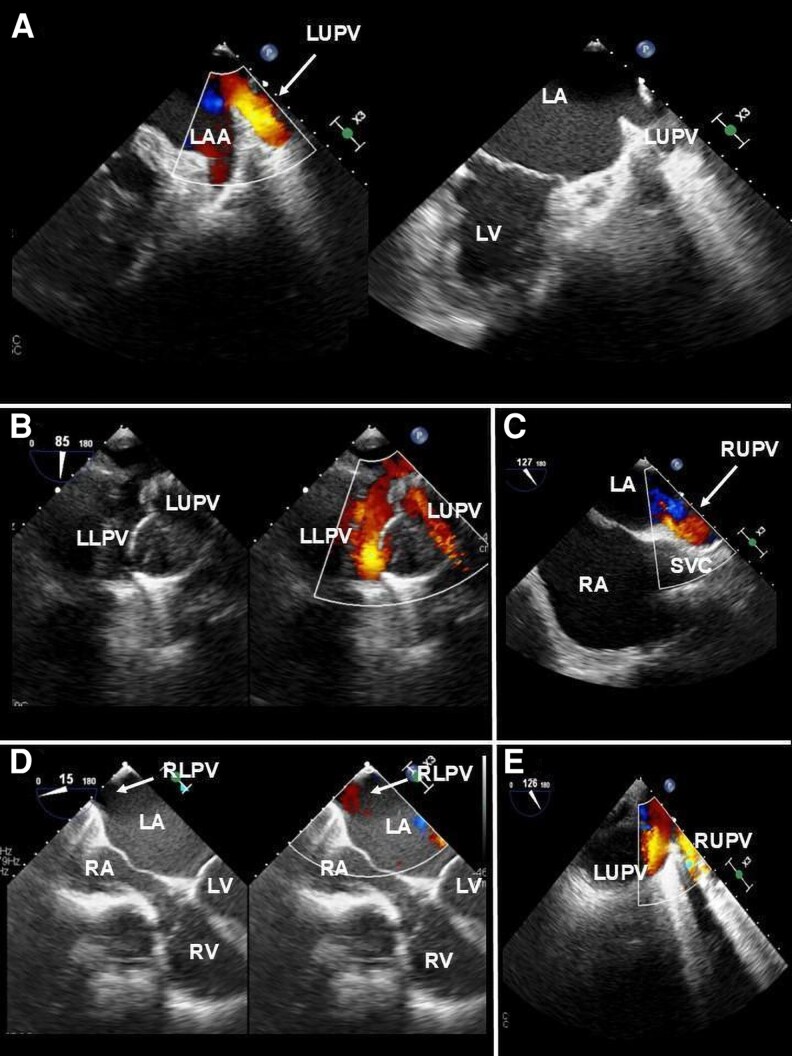
The TOE approach for visualization of PVs requires scanning from the ME level. (*A*): LUPV: from the ME 2-chamber view, the probe is rotated counterclockwise; slight withdrawal and sector rotation back toward ∼60° show the LUPV entering the LA supero-posterior to the LAA (‘LAA view’). (*B*): LLPV: advancing the probe from the LAA view, the LLPV becomes visible once the LAA disappears, with a change in Doppler flow direction. (*C*): RUPV: from the ME bicaval view, counterclockwise rotation of the probe reveals the RUPV entering the LA adjacent to the SVC (‘modified bicaval view’). (*D*): RLPV: from the ME 4-chamber view, clockwise rotation and probe advancement with sector rotation up to 30° demonstrate the RLPV entering the LA from an infero-posterior position. (*E*): RUPV and LUPV: visualized together in a ME long-axis view (∼120°) with extreme counterclockwise rotation, slight probe withdrawal, and probe tilting. LA: Left atrium; LAA: Left atrial appendage; LLPV: Left lower pulmonary vein; LUPV: Left upper pulmonary vein; LV: Left ventricle; ME: Mid-oesophageal; PV: Pulmonary vein; RA: Right atrium; RLPV: Right lower pulmonary vein; RUPV: Right upper pulmonary vein; SVC: Superior vena cava; TOE: Transoesophageal echocardiography.

The following two-dimensional (2D) TOE views can be used: (i) Left Atrial Appendage (LAA) view; (ii) modified left PVs view; (iii) modified bicaval view; (iv) modified right PVs view. Overall, a helpful location aid is the use of colour-flow Doppler with the Nyquist limit set to 40 cm/s (*Figure 1*).

- Left upper pulmonary vein (LUPV): starting from the standard ME 2-chamber view, the probe is turned counterclockwise. Withdrawal of the probe and sector rotation back towards 60° demonstrates the LUPV entering the LA supero-posterior to the LAA (LAA view).
- Left lower pulmonary vein (LLPV): starting from the position described for the LUPV, the probe is advanced towards the atrioventricular junction. The LLPV is visualized once the LAA starts to disappear and is characterized by a change in the direction of Doppler flow. However, visualization is often difficult in this plane.
- LUPV and LLPV: starting from the ME left outflow tract view (120–130°), the probe is turned counterclockwise (modified left PVs view). This view allows for a contemporary scan of both the left-sided veins, with LUPV lying vertically in the lower portion of the sector and LLPV displayed more horizontally in the upper portion of the sector. The LUPV is on the right of the display, and typically the inflow is parallel to the insonation beam, allowing an accurate spectral Doppler assessment.^20^
- Right upper pulmonary vein (RUPV): starting from the standard ME bicaval view, the probe is slightly turned counterclockwise. The RUPV is seen entering the LA adjacent to the SVC (modified bicaval view) (*Figure 1*). Alternatively, starting from the standard ME 4-chamber view, the probe is turned completely clockwise. The use of the colour-flow Doppler reveals the RUPV entering LA from a superior position. The image is optimized by sector rotation up to 30° and by a combination of retroflexion and/or withdrawal of the probe.
- Right lower pulmonary vein (RLPV): starting from the standard ME 4-chamber view, the probe is turned clockwise and advanced towards the atrioventricular junction, with sector rotation up to 30° (*Figure 1*). Colour-flow Doppler aids in the localization of the RLPV entering the LA across the screen from an inferoposterior position.
- RUPV and RLPV: starting from an upper or ME short-axis view (0°–45° sector rotation), the probe is completely turned clockwise. Advancing and/or anteroflexing the probe may aid in the visualization of the vertical scan plane of both right-sided PVs, which usually resemble an inverted Y, with the RUPV having a lower and the RLPV a higher position (modified right PVs view).

3D TOE probes allow the simultaneous viewing of two acoustic windows, at orthogonal planes, by bi-plane imaging mode. Real-time or zoomed volume rendering 3D is challenging for the visualization of non-stented PVs, due to low spatial resolution and signal void, however it can be facilitated when stenting has been performed.

**Figure 2:**
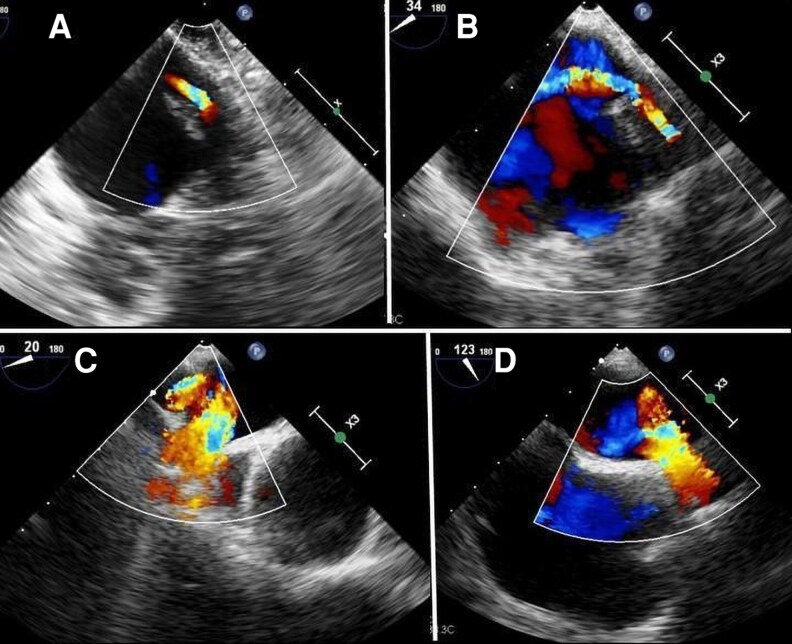
TOE is useful for identifying and confirming PV stenosis and for estimating pressure gradients. Colour Doppler with a low-velocity scale (< 40 cm/sec) enhances PV visualization: turbulent flow across the stenotic tract generates a mosaic pattern due to acceleration and aliasing. (*A*): 2D-colour-Doppler of the LUPV (from LAA view) showing aliasing consistent with stenosis. (*B*): 2D-colour-Doppler of the LUPV demonstrating multicolour aliasing due to increased flow velocity from multiple stenosis. (*C*): 2D-colour-Doppler of the RLPV (from a superior ME short-axis view) showing turbulent flow (*D*): 2D-colour-Doppler of the RUPV (from the ME modified bicaval view) with aliasing related to stenosis. LAA: Left atrial appendage; LUPV: Left upper pulmonary vein; ME: Mid-oesophageal; PV: Pulmonary vein; RLPV: Right lower pulmonary vein; RUPV: Right upper pulmonary vein; TOE: transoesophageal echocardiography.

**Figure 3:**
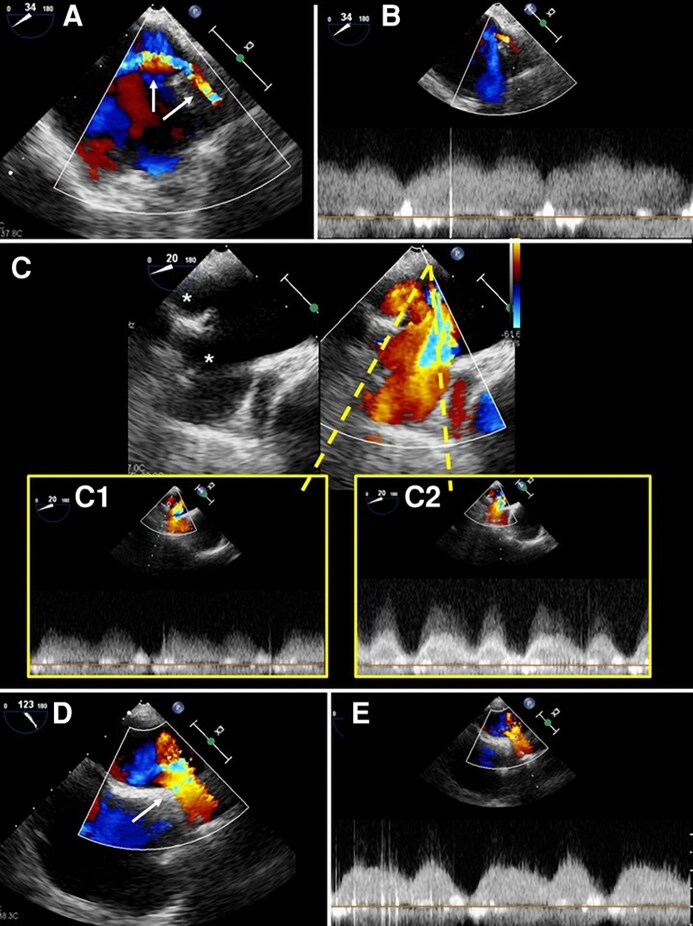
Flow acceleration and gradients across PV stenosis are assessed by positioning the pulsed-wave (PW) Doppler sample volume within the centre of the vein or at the level of maximal aliasing. Normally, pulmonary vein PW Doppler shows three waves: systolic (S), diastolic (D), and atrial reversal (Ar). Stenosis is considered significant when the maximum PV flow velocity exceeds 1.1 m/s. (*A*): 2D colour Doppler of the LUPV showing multicolour aliasing (white arrows) due to stenosis. (*B*): PW Doppler interrogation of the LUPV demonstrates high-velocity flow consistent with stenosis. (*C*): PW Doppler interrogation of the right PVs (asterisks), with higher velocities/gradient in panel C.2 compared with panel C.1. (*D*): 2D colour Doppler of the RUPV (from the ME modified bicaval view) showing aliasing (white arrow) due to stenosis. (*E*): PW Doppler recording with blunted systolic velocity and prolonged elevated diastolic velocity, with severe stenosis. LUPV: Left upper pulmonary vein; ME: Mid-oesophageal; PV: Pulmonary vein; RUPV: Right upper pulmonary vein.

Application of colour-Doppler mode, using a low-velocity scale (< 40 cm/sec), aids in the identification of the PVs: while the absence of stenosis is characterized by laminar, continuous (systolic and diastolic) red flow, systolic reverse flow, due to significant mitral regurgitation, is displayed as a blue component entering the PVs. With PVS, the turbulent flow crossing the stenotic tract produces a mosaic of colours, due to the acceleration and the aliasing effect at colour-Doppler (*Figure 2*). Quantitative assessment of flow acceleration and gradients across PVS requires placing the pulsed wave (PW) sample-volume box > 0.5 cm within the centre of the vein or at the level of maximal aliasing (*Figure 3*). A normal pulmonary vein spectral Doppler trace has three waves: the systolic (S) and diastolic (D) waves, caused by antegrade ventricular systolic and diastolic flow and seen above the baseline, and the atrial systolic reversal (AR) wave, caused by retrograde atrial systolic flow, and seen below the baseline. Normal velocities of S-wave, D-wave, and AR-wave are 30–80 cm/s, 20–70 cm/s, and 10–25 cm/s, respectively. Increased peak pulmonary vein velocities (>1.1–1.5 m/s) and turbulent flow at the pulmonary vein ostium may suggest haemodynamically significant stenosis.^21^ However, Doppler findings should be interpreted with caution, as measured velocities are influenced by several factors, including flow volume, LA pressure, and the presence of multivessel disease. Moreover, Doppler assessment is inherently angle-dependent and may be affected by confounding conditions such as mitral regurgitation. Therefore, echocardiographic findings should always be integrated with anatomical imaging, particularly contrast-enhanced CT angiography.

## Planning

**Figure 4:**
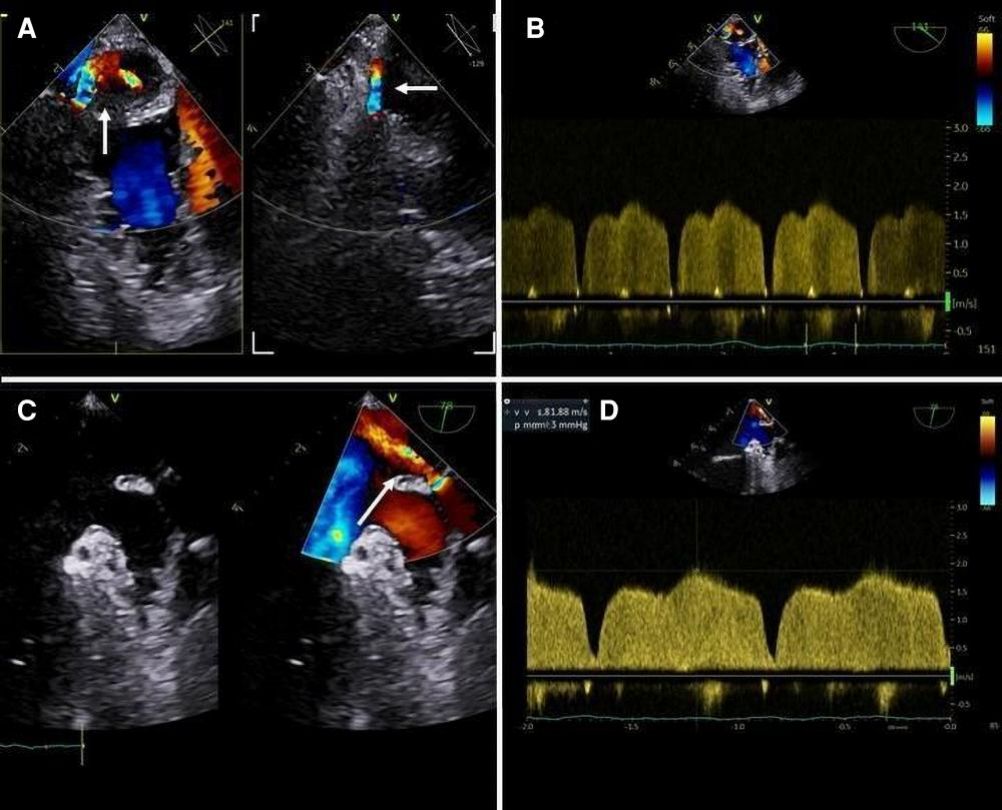
TOE is crucial for identifying multiple PV stenoses, which guides procedural planning, including the number and location of stents. In patients with >1 PV affected, a staged approach, during the same procedure or at separate sessions, is generally recommended, beginning with the most severe stenosis. Stent sizing is defined preoperatively by non-invasive imaging. (*A*) Biplane colour Doppler of the left PVs (from the LAA view) showing aliasing due to stenosis (white arrows). (*B*) PW Doppler interrogation of the LUPV demonstrating high-velocity flow (2 m/s). (*C*) 2D and colour Doppler interrogation of the LLPV showing aliasing (white arrow). (*D*) PW Doppler interrogation of the LLPV reveals flow acceleration with lower velocity than LUPV (1.6 m/s). LAA: left atrial appendage; LLPV: left lower pulmonary vein; LUPV: left upper pulmonary vein; PV: pulmonary vein; PW: pulsed wave; TOE: transoesophageal echocardiography.

Analysis of TOE images in colour-Doppler mode may lead to the identification of multiple acceleration sites of PV flow, due to multiple stenosis (*Figures 3* and *4*). The proper identification of this condition allows to plan the number of stents to be positioned, their location, and the possible need for post-implantation balloon-dilation. Generally, patients with stenosis in more than one PV should undergo a staged approach (during the same procedure or at different times), starting from the most severe PVS.

The choice of the stent size is performed pre-operatively by analysing non-invasive imaging.

## Step-by-step procedural guidance

In the cath lab, TOE plays a key role during all the stages of the procedure.

**Figure 5:**
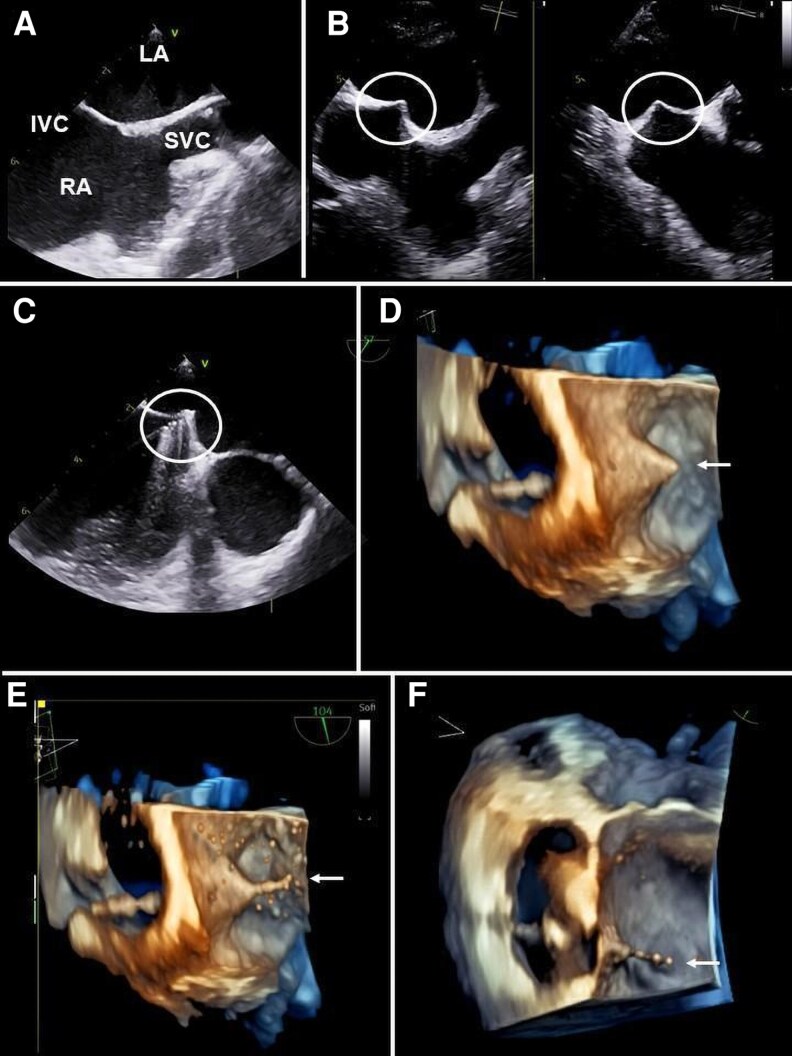
TOE plays a pivotal role in ensuring safe and accurate transseptal access. (*A*) Standard ME bicaval view showing the catheter-guide advancing from the IVC towards the fossa ovalis and the needle approaching the septum; proper identification of the fossa ovalis is crucial. (*B*) Biplane imaging enables accurate positioning and orientation of the catheter tip. (*C*) Needle pressure on the fossa ovalis procedures the typically ‘tenting’ sign (white circle). (*D*) 3D zoom en-face view of the fossa ovalis from the RA facilitates manipulation of the dilator; the white arrow indicates IAS tenting produced by the transseptal sheath. (*E* and *F*) Once adequate tenting is obtained, 3D zoom confirms correct and stable passage of the electrocautery system and wire across the fossa ovalis (white arrows). IAS: interatrial septum; IVC: inferior vena cava; LA: left atrium; ME: mid-oesophageal; RA: right atrium; SVC: superior vena cava; TOE: transoesophageal echocardiography.

**Figure 6:**
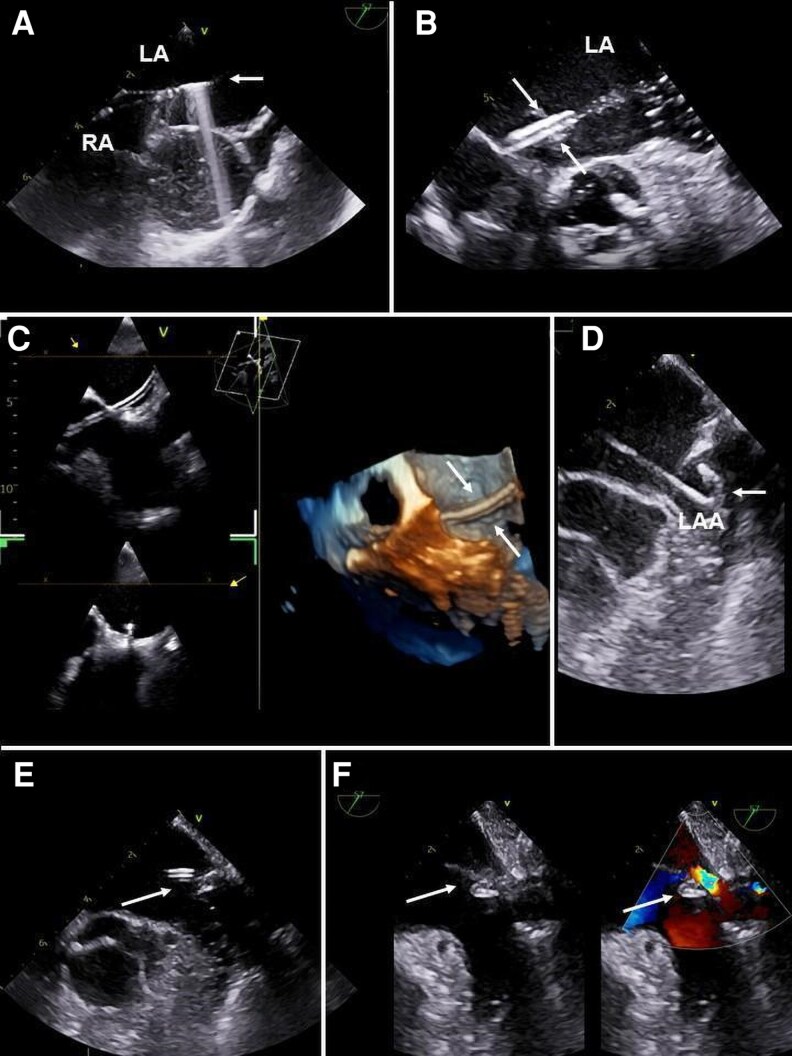
After transseptal access, wires and catheters are advanced into the LA under continuous TOE monitoring, avoiding contact with the atrial wall, aortic wall, or LAA lumen. Once the guidewire reaches the target stenosed PV, the delivery sheath is positioned at its ostium. (*A*) 2D TOE ME bicaval view showing passage of the wire (white arrow) from the RA to the LA. (*B*) 2D TOE superior ME short-axis view demonstrating the catheter (double white arrows) within the LA. (*C*) 3D reconstruction confirming catheter position (double white arrows) in the LA. (*D*) LAA view showing incorrect catheter allocation into the LAA (white arrow). (*E*) LAA view showing correct catheter orientation (white arrow) towards LUPV. (*F*): Simultaneous 2D and colour Doppler view of the LUPV confirming catheter placement (white arrow) within the stenotic LUPV. LA: left atrium; LAA: left atrial appendage; LUPV: left upper pulmonary vein; ME: mid-oesophageal; PV: pulmonary vein; RA: right atrium; TOE: transoesophageal echocardiography.

**Figure 7:**
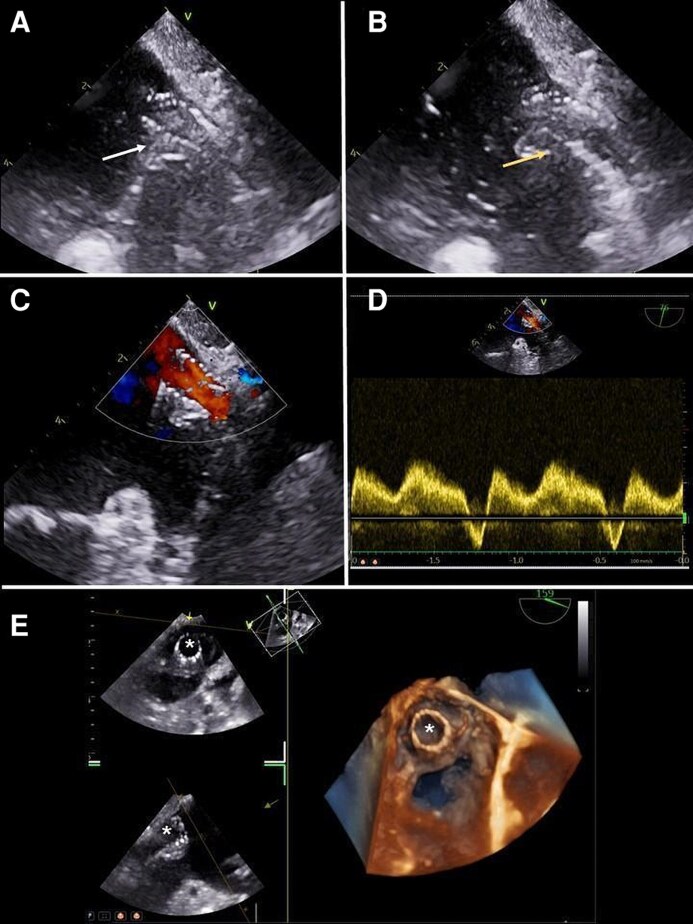
TOE is essential for guiding PV dilatation and stent deployment. After each dilatation, mean pressure gradients must be measured and confirmed by venous angiography. (*A*) An inflated balloon is clearly visualized during dilatation (white arrow). (*B*) After deflation, the hyper-echoic stent structure is visible within the PV (yellow arrow). (*C*) Colour Doppler is used to exclude residual flow acceleration requiring further dilatation, either within the stent, at side branches, or distal to the stent. (*D*) After each dilatation, mean pressure gradient is assessed by both PW Doppler and catheterization to confirm procedural success. (*E*) Real-time or zoomed 3D LA view displaying stent edges protruding at the ostium into the atrial chamber, without impingement on surrounding structures (asterisks). A: left atrium; PV: pulmonary vein; PW: pulsed wave.

**Figure 8:**
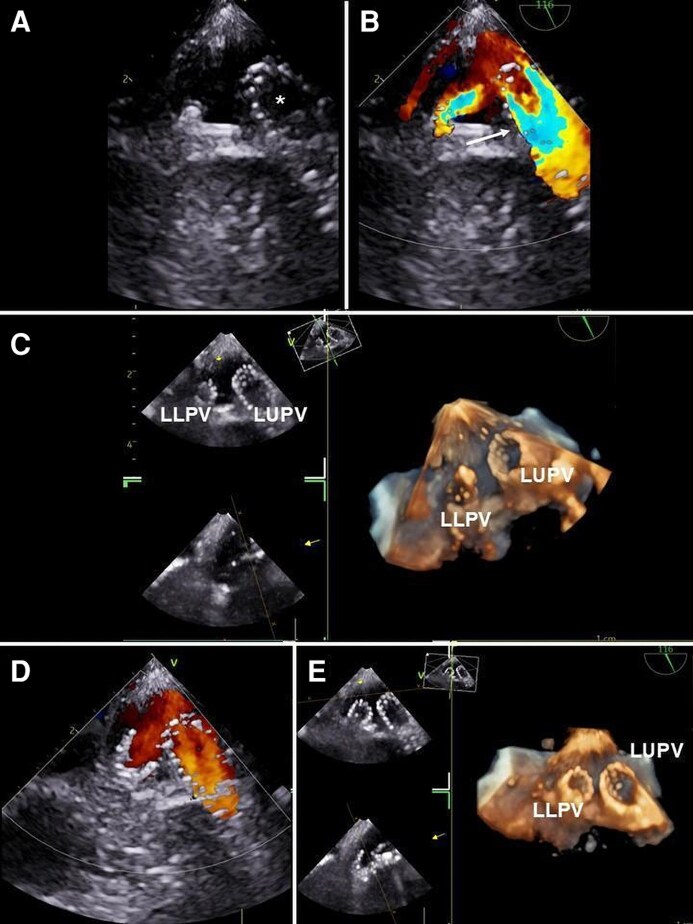
After balloon dilatation or stent placement, TOE is essential not only for assessing procedural success (defined as a mean pulmonary vein gradient <2 mmHg and residual stenosis <20%) but also for detecting residual stenosis. In addition, TOE allows evaluation of the immediate haemodynamic effects, including biventricular function and pulmonary artery pressure, and the identification of potential complications such as pericardial effusion or residual interatrial septal defect. (*A*) Stent implanted into the LUPV (asterisk). (*B*) 2D colour Doppler of the LUPV after stent implantation showing aliasing within a dilated vein (white arrow). (*C*) 3D real-time view of stent implanted in the LUPV and stent dilatation in the LLPV. (*D*) 2D colour Doppler of the left PVs post-stents implantation showing laminar flow across the vessels. (*E*) 3D en-face view of the stents implanted in the left PVs. LLPV: left lower pulmonary vein; LUPV: left upper pulmonary vein; PV: pulmonary vein; TOE: transesophageal echocardiography.

1. ***Selecting the site for trans-septal puncture***. Firstly, TOE is of utmost importance during *trans*-septal puncture. The standard bicaval view is the TOE view of choice for starting the procedure, as it allows for the visualization of both the catheter-guide coursing from the inferior vena cava (IVC) to the fossa ovalis and the needle approaching it (*Figure 5*). Proper identification of the fossa ovalis and conditions with increased puncture risk (small dimensions or aneurysmal or thickened fossa ovalis, increased thickness of septum secundum, evidence of patent foramen ovale, the presence of surgical suture resulting from biatrial *trans*-septal approach in previous heart surgery) is essential. Sometimes, after entering the right atrium (RA), rapid formation of thrombotic masses attached to the catheter and floating within the RA, may occur: in this condition, it is necessary to obtain a resolution of thrombi by anticoagulation before crossing the IAS. Usually, after reaching RA from the IVC, the catheter delivering the needle is first allocated within the SVC. From this position, it is slowly retracted by impinging IAS until the exact point of puncture is reached. Simultaneous biplane visualization (bicaval and short axis) allows to achieve the correct position and orientation of the tip of the catheter (*Figure 5*). This ensures that the catheter is anteriorly orientated and that it is not engaging PFO, which increases the risk of aortic perforation. 3D imaging with a zoom en-face perspective view of fossa ovalis from RA may help moving the dilator within the fossa ovalis. The preferred site of puncture for approaching PVs is the inferior and anterior portion of fossa ovalis. The position of the needle within the fossa ovalis is usually identified through the recognition, at 2D imaging, of the typical tenting under the pressure of the tip of the needle (*Figure 5*). If the needle does not produce adequate tenting, clockwise or counterclockwise rotation of the probe or the use of 3D imaging, in the biplane perspective, may be helpful, as it can be located outside the 2D scan plane.
2. ***Guiding and monitoring transeptal puncture.*** When a safe position with adequate tenting has been achieved, simultaneous multiplane view (bicaval and short axis) or 3D zoom allows for the visualization of the correct and stable passage of the needle across the fossa ovalis and then of the dilator and the sheath. Real-time or zoomed 3D modes display the passage of the needle across the fossa ovalis (*Figure 5*).
3. ***Monitoring wire, catheter crossing the target vein lesion***. The next steps include advancing wires and catheters within LA and avoiding the delivery systems and materials pushing against atrial wall, aortic wall, or LAA lumen, under constant TOE visualization (*Figure 6*). Puncture of these structures may produce immediate pericardial effusion with cardiac tamponade. Once the guidewire has targeted the stenosed PV, a delivery sheath is allocated within the ostium of the target PVs. An invasive measure of the mean pressure gradient across the lesion is performed and then contrast-enhanced venography is carried out to confirm the stenosis. This requires the use of contrast medium and radiation and obtaining a real-time guide can be difficult. TOE offers potential advantages over fluoroscopic guidance, as it is less invasive and provides real-time 2D multiplane views or 3D images, which allow for the safe guidance of each procedural step. Thus, taking care about the distal position of the wires, a safe crossing of the target lesion can be achieved, avoiding pulmonary vein perforation by the wire, which is one of the most frequent procedural complications.
4. ***Monitoring balloon dilatation and stent positioning***. TOE is also useful in guiding balloon dilation and stent positioning: the inflated balloon is clearly visualized during dilatation, and once it is deflated, hyper-echoic stent structure appears within the PV (*Figures 7* and *8*). Currently, colour-Doppler mode is used for ruling out possible residual acceleration sites needing further balloon dilation along the stent, or at the level of side branches or eventually distal to the stent. Moreover, after each dilatation, measuring the mean pressure gradient is mandatory: a successful procedure is defined as mean pulmonary gradient < 2 mmHg and a residual stenosis < 20%.^19^ The results need to be confirmed by venous angiography. Real-time or zoomed 3D modes from the LA perspective display the stent edge protruding over their ostia within the atrial chamber, without impingement of surrounding structures.
5. ***Check the result***. After ballooning or stent positioning, TOE is useful for evaluating the immediate haemodynamic effects of the intervention, for analysing right and left ventricular function and pulmonary artery pressure, as well as for evaluating possible complications (excluding pericardial effusion and evaluating the residual interatrial septum defect).

## Follow-up

Although acute procedural success rates generally exceed 90%, restenosis remains a recognized limitation.^11^ A structured multimodality imaging follow-up is therefore recommended. TTE represents a useful non-invasive tool for initial surveillance, while contrast-enhanced CT is considered the reference modality for the assessment of pulmonary vein restenosis. In selected cases, TOE may provide additional haemodynamic information through Doppler evaluation of pulmonary vein flow. Although no universally accepted follow-up protocol exists, several authors have proposed serial imaging during the first year after intervention, typically including CT angiography at 3–6 months and again at 12 months, particularly in symptomatic patients or when echocardiographic findings raise suspicion of restenosis.^14^

## Conclusions

Pulmonary vein stenosis is an uncommon but potentially serious complication following atrial fibrillation ablation that requires a high index of clinical suspicion and a multimodality imaging approach for accurate diagnosis and management.

While cross-sectional imaging plays a key central role in anatomical characterization, echocardiography—particularly TOE—provides essential haemodynamic information and real-time guidance during percutaneous intervention.

The step-by-step TOE-guided workflow described in this report highlights the practical value of echocardiography across the entire clinical pathway, from diagnosis to procedural guidance and post-intervention assessment.

The integration of multimodality imaging within a structured procedural approach may improve procedural planning, facilitate safe pulmonary vein recanalization, and optimize post-procedural surveillance.

## Data Availability

No new data were generated or analysed in support of this research.
